# Functional Validation of the Putative Oncogenic Activity of *PLAU*

**DOI:** 10.3390/biomedicines11010102

**Published:** 2022-12-30

**Authors:** Federica Sarno, Désirée Goubert, Emilie Logie, Martijn G. S. Rutten, Mihaly Koncz, Christophe Deben, Anita E. Niemarkt, Lucia Altucci, Pernette J. Verschure, Antal Kiss, Wim Vanden Berghe, Marianne G. Rots

**Affiliations:** 1 Epigenetic Editing, Department of Pathology and Medical Biology, University Medical Centre Groningen, University of Groningen, Hanzeplein 1, 9713 GZ Groningen, The Netherlands; 2 Center for Oncological Research (CORE), Integrated Personalized & Precision Oncology Network (IPPON), Laboratory of Protein Chemistry, Proteomics and Epigenetic Signalling (PPES), Department of Biomedical Sciences, University of Antwerp, 2610 Wilrijk, Belgium; 3 Department of Paediatrics, University Medical Centre Groningen, University of Groningen, Hanzeplein 1, 9713 GZ Groningen, The Netherlands; 4 Swammerdam Institute for Life Sciences, University of Amsterdam, Science Park 904, 1098 XH Amsterdam, The Netherlands; 5 Institute of Biochemistry, Biological Research Centre, H-6726 Szeged, Hungary; 6 Doctoral School of Biology, Faculty of Science and Informatics, University of Szeged, H-6726 Szeged, Hungary; 7 Center for Oncological Research (CORE), Integrated Personalized & Precision Oncology Network (IPPON), University of Antwerp, 2610 Wilrijk, Belgium; 8 Department of Precision Medicine, Universita’ degli Studi della Campania “Luigi Vanvitelli”, 80138 Naples, Italy; 9 Biogem Institute of Molecular and Genetic Biology, 83031 Ariano Irpino, Italy; 10 IEOS Istituto per l’Endocrinologia e l’Oncologia “Gaetano Salvatore”, Via Pansini, 80131 Napoli, Italy

**Keywords:** Epigenetic Editing, CRISPR-dCas9, triple negative breast cancer, *PLAU*, oncogene

## Abstract

Plasminogen activator, urokinase (*PLAU*) is involved in cell migration, proliferation and tissue remodeling. *PLAU* upregulation is associated with an increase in aggressiveness, metastasis, and invasion of several cancer types, including breast cancer. In patients, this translates into decreased sensitivity to hormonal treatment, and poor prognosis. These clinical findings have led to the examination of *PLAU* as a biomarker for predicting breast cancer prognosis and therapy responses. In this study, we investigated the functional ability of *PLAU* to act as an oncogene in breast cancers by modulating its expression using CRISPR-deactivated Cas9 (CRISPR-dCas9) tools. Different effector domains (e.g., transcription modulators (VP64, KRAB)) alone or in combination with epigenetic writers (DNMT3A/3L, MSssI) were fused to dCas9 and targeted to the *PLAU* promoter. In MDA-MB-231 cells characterized by high *PLAU* expression downregulation of *PLAU* expression by CRISPR-dCas9-DNMT3A/3L-KRAB, resulted in decreased cell proliferation. Conversely, CRISPR-dCas9-VP64 induced *PLAU* upregulation in low *PLAU* expressing MCF-7 cells and significantly increased aggressiveness and invasion. In conclusion, modulation of *PLAU* expression affected metastatic related properties of breast cancer cells, thus further validating its oncogenic activity in breast cancer cells.

## 1. Introduction

Plasminogen activator, urokinase (*PLAU*) also known as urokinase-Type Plasminogen Activator (u-PA), encodes a protease that converts inactive plasminogen to the active serine protease plasmin [1]. Plasmin is involved in the breakdown of the extra-cellular matrix (ECM), and hence, plays an important role in favoring cell migration, cell proliferation and tissue remodeling. Based on the increased risk of metastasis that is associated with these functions, the oncogenic role of *PLAU* in several cancers, including breast cancer, has long been suggested [1,2,3,4,5,6,7]. In different studies it was shown that experimental inhibition of *PLAU* reduced the tumor growth, the aggressiveness and metastasis [1,8,9,10]. In one example, mice were inoculated with murine Lewis lung carcinoma cells and treated with anti-human antibodies directed towards *PLAU*. These antibodies cross-react with the murine *PLAU* and thereby inhibit *PLAU* activity. Injections with these antibodies resulted in significant inhibition of lung metastasis in a dose-dependent matter [5]. In a different study using a similar method, nude mice were inoculated with human squamous carcinoma cells, which express *PLAU* at high level and are known to metastasize to lungs and lymph nodes. Injection of these mice with anti-human *PLAU* antibodies abolished local invasion of the tumors [4]. Similarly, suppression of *PLAU* in human ovarian cancer cells by antisense phosphorothioate oligonucleotides delivered by liposomes, showed a reduced invasive capacity of the tumor when compared to untreated cells, and a reduction in intraperitoneal spread of the cancer cells in nude mice treated with *PLAU* antisense phosphorothioate oligonucleotides was also observed [6].

In breast cancer patients, increased levels of *PLAU* were associated with a worse prognosis and an increase in aggressiveness, metastasis, and invasion [11,12,13,14,15]. These clinical findings have led to the investigation of *PLAU* as a biomarker for predicting breast cancer prognosis and responsiveness to hormonal agents such as endocrine therapy [16,17,18]. Upfront determination of *PLAU* levels could help to classify patients, also of multiple other cancer types, for their risk for metastasis and treatment relapse, and thus to assist clinicians in designing individualized treatment strategies [11,12,13,14,19].

Since selective inhibition of *PLAU* is not yet clinically available, other methods to inhibit *PLAU* have been investigated [20,21,22,23]. One promising phytochemical compound is Withaferin A (WA). WA is a natural compound with wide-ranging pharmacological activities including cardio-protective, anti-inflammatory, immuno-modulatory, anti-angiogenesis, anti-metastasis and anti-carcinogenic effects [24,25,26]. WA decreases expression of genes encoding ECM-degrading proteases (including *PLAU*), as well as the expression of other genes involved in cell adhesion, inflammation, and metastasis in vitro and in vivo [20,24,27,28,29]. Treatment of the hormone-insensitive, aggressive MDA-MB-231 triple negative breast cancer cell line with WA led to a decreased *PLAU* expression and reversed its highly invasive metastatic phenotype. In fact, upon WA treatment, MDA-MB-231 cells displayed characteristics similar to those of the non-invasive, hormone-sensitive, estrogen receptor α positive luminal A MCF-7 breast cancer cells, which are derived from a milder and more treatable breast cancer subtype [20]. Moreover, Szarc vel Szic et al. found that WA treatment of MDA-MB-231 cells promoted *PLAU* promoter DNA hypermethylation resulting in phenotypes similar to those of the less aggressive luminal B stage breast cancer type [28]. Indeed, WA changed expression levels of several epigenetic DNA/histone methylation enzymes, which subsequently leads to reduced H3K4 me2/me3 histone methylation levels of WA-responsive genes, including *PLAU*, potentially explaining the observed changes in gene expression and phenotype [30,31,32]. Pakneshan et al. described how the methylation status of the *PLAU* promoter can be used as a prognostic marker in patients with breast carcinoma [33]. Since *PLAU* demethylation correlates with a bad prognosis [34], targeted methylation of *PLAU* is proposed as a potential novel therapy [35]. Guo Y et al., showed that methylation of the *PLAU* promoter inhibited Ets-1 transcription factor binding, thus blocking its transcription. In the same study it was shown that DNA methylation is the dominant mechanism involved in silencing *PLAU* gene expression [34]. Chik et al., investigated the effect of 5-azacytidine in combination with a DNA-methylating agent, showing that 5- azacytidine on its own could induce demethylation of pro-metastatic genes as well [35]. Technologies to induce gene-specific methylation of the *PLAU* target gene could thus present a solution to this problem. These observations prompted us to use CRISPR-dCas9 (deactivated CRISPR-Cas9) based Epigenetic Editing strategies for gene specific control of *PLAU* expression.

CRISPR-dCas9 can be coupled to epigenetic enzymes (“Epigenetic Editing”) and targeted specifically to modulate the expression of genes of interest [36,37,38]. Importantly, numerous in vivo approaches have indicated therapeutic potency [37,38,39]. By guiding the correct combinations of different effector domains (EDs), i.e., DNA and histone methylation state editors to the gene of interest, stable modulation of gene expression can be achieved [40,41,42,43,44]. Recently, Nunez et al. demonstrated that dCas9 fused to DNMT3A/L and KRAB (CRISPRoff-v2.1) led to long-lasting gene silencing for a wide spectrum of genes [45]. In addition to silencing resistance genes and modulating immune responses, long-lasting repression of genes involved in cancer progression by Epigenetic Editing might provide a novel approach to cancer therapy. In this study our aim was to test the oncogenic activity of *PLAU* in breast cancer cells, and to obtain further insight into *PLAU* as a potential therapeutic target in oncology.

## 2. Materials and Methods

### 2.1. Cell Culture and Chemical Reagents

Human embryonic kidney cells HEK293T (ATCC: CRL-3216), human estrogen receptor positive breast cancer cells MCF-7 (ATCC: HTB-22) and triple negative human breast cancer cells MDA-MB-231 (ATCC: CRM-HTB-26) were all cultured in DMEM medium (Lonza BioWhittaker #BE12-604F) supplemented with 10% fetal bovine serum (FBS), 2 mM L-glutamine and 50 μg/mL gentamycin sulfate. Cells were cultured in a humidified incubator with 5% CO_2_ at 37 °C. All cells were routinely tested for mycoplasma contamination.

### 2.2. Plasmid Construction

#### 2.2.1. Plasmids Encoding Transiently Expressed dCas9-Effector Domains

Plasmids pMLM3705 (dCas9-VP64) and MLM3636 (sgRNAs) were kind gifts from Keith Joung (Addgene plasmid #47754 and #43860, respectively), and CRISPRoff-v2.1 (OFF2.1) (DNMT3A/3L-dCas9-KRAB) from Jonathan S Weissman [45] (Addgene Plasmid #167981). We used dCas9-NED (no effector domain) (Addgene plasmid #109358) as negative control [46]. The catalytic domain of human histone methyltransferase PRDM9 was amplified from total cDNA of a testicular cancer cell line, and the catalytic domain of the histone methyltransferase DOT1L from human fibroblasts by Pfu DNA polymerase (Thermo Scientific, Leon-Rot, Germany) as described elsewhere [41]. These catalytic domains were inserted into dCas9-NED to create dCas9-PRDM9 and dCas9-DOT1L. The enzymatically inactive mutants were obtained by site-directed mutagenesis (Table S2).

#### 2.2.2. Single Guide RNAs (sgRNAs)

**Figure 1:**
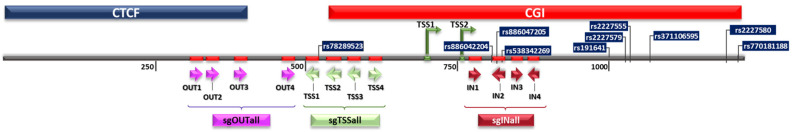
Position of sgRNAs designed to modulate *PLAU* gene expression.

Several sgRNAs targeting different regions of the *PLAU* gene (Figure 1) were cloned by inserting DNA oligonucleotides containing the 20-bp target region between the two BsmBI sites of MLM3636 [46]. As negative control, MLM3636, for transient transfection, or sgOUT(1–4), for lentiviral transduction, without inserted target sequence were used together with the effector domains (empty vector, EV). The sgOUT(1–4) plasmid was created to contain four sgRNA sequences (sgOUTall) in one plasmid. The MLM3636 plasmid was first digested with Acc65I to remove the original sgRNA expressing cassette. After self-re-circularization we had an empty vector which was digested with NheI and BamHI restriction enzymes. Additionally, the lenti sgRNA zeo backbone (Addgene#61427) was also digested with NheI and BamHI to cut out the coding sequence for sgRNA2.0. By ligating sgRNA2.0 with the digested empty vector plasmid a new plasmid (pMLM2.0) was obtained. To allow expression of multiple guides, an oligoduplex (AK497 and AK498) encoding restriction sites for StyI and Acc65I, respectively, (also restriction sites for BveI enzymes, which results in homologues ends after digestion with StyI and Acc65I) was inserted between the BamHI and Acc65I sites after the sgRNA2.0 sequence (pMLM2.0_T). Double digestion with StyI and Acc65I or with BveI results in compatible ends with NheI and Acc65I digested fragments. These restriction sites allowed us to sequentially clone different sgRNA expressing cassettes into one plasmid. The sgRNA tandem plasmid was created by inserting the 447-length NheI-Acc65I fragment encoding the sgRNA expressing cassette into pMLM2.0_T plasmid digested with StyI and Acc65I or BveI.

The plasmid lenti sgRNA (MS2) zeo backbone was a kind gift from Feng Zhang (Addgene plasmid # 61427). All four sgOUTall were cloned and inserted into the lentiviral sgRNA backbone in exactly the same way as for the transiently expressing MLM3636 sgRNA plasmid, by inserting DNA oligonucleotides containing the 20-bp target region between the two BsmBI sites of the lentiviral sgRNA plasmid (Table S2).

#### 2.2.3. Lentiviral dCas9-Effector Domains

The lentiviral expression plasmid vector pHAGE EF1α dCas9-VP64 was a gift from Rene Maehr and Scot Wolfe (Addgene plasmid #50918). To create several stable cell lines, dCas9-EDs were made by replacing the VP64 gene with an oligonucleotide containing a MluI and an AsiSI site. By using AscI-PacI double digestion on previously obtained dCas9-EDs and transferring the EDs into pHAGE EF1α dCas9-NED, we created pHAGE EF1α dCas9-ED [46]. Briefly, pHAGE EF1α dCas9-M.SssI-Q147L and pHAGE EF1α dCas9-M.SssI-E186A were constructed by cutting out the M.SssI-Q147L and M.SssI-E186A genes with SgsI (AscI) and PacI restriction enzymes and cloning them in the pHAGE EF1α dCas9-NED vector between the AsiSI and MluI sites. The G9a catalytic domain and its mutant were digested out from pMX-ZF-IRES-GFP [47] with MluI and NotI and subcloned into the pHAGE EF1α dCas9- VP64, in which an additional multiple cloning site was added by replacing the coding sequence of the VP64 activator domain with a sequence containing a MluI restriction site. The Super KRAB (SKD) domain was subcloned by amplifying with primers containing MluI and NotI overhangs (Table S2).

### 2.3. Creation of Stable Cell Lines

Creation of MCF-7 or MDA-MB-231 cell lines stably expressing dCas9-ED was described elsewhere [46]. Briefly, lentiviral pHAGE-EF1α constructs encoding the dCas9-EDs were co-transfected with the second-generation packaging plasmids pCMVΔR8.91 and pCMV-VSV-G (#8454, Addgene, Watertown, MA, USA) on day one into HEK293T cells using PEI transfection reagents (#23966, Polysciences, Inc., Warrington, PA, USA) to produce lentiviral particles. The supernatant of HEK293T cells containing the virus was harvested 48 and 72 h after transfection. Host cells (MCF-7 or MDA-MB-231) were seeded in six-well plates and transduced on two consecutive days (day three and four) with 1.5 mL of the viral supernatant, supplemented with 8 µg/mL polybrene (Sigma-Aldrich, St. Louis, MO, USA). The transduced cells were selected on day seven in 8 µg/mL puromycin-supplemented medium for four days and subsequently cultured in 1 µg/mL puromycin-supplemented medium.

### 2.4. Lentiviral Transduction of Cells

For the lentiviral transductions of sgRNAs in stable MDA-MB-231 cells, the lentiviral sgRNA were co-transfected with the second-generation packaging plasmids pCMVΔR8.91 and pCMV-VSV-G on day one into HEK293T cells (seeded the previous day to reach a confluency of 70%) using PEI transfection reagents (#23966, Polysciences, Inc.) to produce lentiviral particles. The supernatant of HEK293T cells containing the virus was harvested at 48 and 72 h after transfection, supplemented with 8 µg/mL polybrene (Sigma-Aldrich) and frozen at −20 °C for a maximum of one month. MDA-MB-231 stably expressing dCas9-ED cell lines were seeded in normal medium (not supplemented with puromycine) on a 6-well plate at a concentration that would ensure 70% confluency on the next day. The viral supernatant that was harvested after 48 h, was thawed, supplemented again with 8 µg/mL polybrene and FBS and 1.5 mL of the pre-warmed and supplemented viral supernatant was applied to the stably expressing MDA-MB-231 cells at 24 h after seeding. After 12 h the medium containing the viral particles was refreshed with the viral supernatant that was harvested after 72 h, and prepared in the same way as before. The transduced cells were collected at 48 h after the first transduction (12 h after the second transduction) to assess their effect on gene expression.

### 2.5. Transient Transfection of Cells

Cells were seeded at a concentration that would ensure 70% confluency on the next day (day of transfection). MDA-MB-231 cells were plated in 24 well plate (1 × 10^5^ cells/well), and transfected with 0.75 μg plasmid DNA (375 ng sgRNAs and 375 ng dCas9-ED) using 1.5 μL of Attractene Transfection Reagent (#301005, Qiagene, Venlo, The Netherlands). HEK293T and MCF-7 (5 × 10^5^ cells/well), plated in 6-well plates, were transfected with 1 μg DNA (500 ng sgRNAs and 500 ng dCas9-ED) using SAINT (Synvolux Products & Therapeutics, Leiden, The Netherlands) in a 2:1 ratio for the wild-type cells or PEI in a 4:1 ratio for the stable cell lines. Twenty-four hours after transfection the medium was changed to normal growth medium, and 48 h after transfection cells were harvested to assess the short-term effect on gene expression. All transient transfection experiments were performed in triplicate.

To assess long-term effects on gene expression and proliferation capacity, cells were cultured for 5 or 12 days after transfection, and split when 80–90% confluent. Transient expression of sgRNAs allowed assessment of long-term effects on gene expression because the plasmids faded out with each cell division, even if the dCas9-ED was stably expressed (as in the case of the stable cell lines).

### 2.6. Quantitative Real-Time PCR

Total RNA was isolated using TRIzol reagent (Thermo Scientific, Waltham, MA, USA) according to the manufacturer’s protocol. Subsequently, cDNA was generated using the Revertaid cDNA synthesis kit with random hexamer primers (Thermo Scientific) or with M-MLV Reverse Transcriptase (Promega, Madison, WI, USA) and oligo (dT) primers. To assess gene expression, qRT-PCR was performed with 10 ng of cDNA input, using ABsolute qPCR SYBR Green (Thermo Scientific) or GoTas Green master Mix (Promega). *PLAU* expression was normalized to *GAPDH*, a house keeping gene, and all reactions were performed in triplicate using an ABI ViiA7 real-time PCR system (Applied Biosystems, Waltham, MA, USA) for 45 cycles or Rotor-Gene Q (Qiagen). Primer sequences are provided in Table S1. Ct values were obtained, and quantitative analysis was performed using the cycle threshold (ΔΔCt) method after normalization to *GAPDH* expression. Fold change was calculated relative to control samples.

### 2.7. Migration Assay

The migration capacity of cells was evaluated using the xCELLigence real-time cell analysis (RTCA) system (Roche, Penzberg, Germany) as previously described [20]. Briefly, 160 µL and 30 µL of media was added to the lower and upper chambers of modified 16-well plates (CIM-16, Roche), respectively. The lower chambers either contained FBS supplemented or FBS free medium to assess chemotactic and background migration. CIM-16 plates were subsequently placed in the RTCA DP instrument at 37 °C for 1 h to measure background signal. Serum-deprived stable MCF-7 cells were harvested using TrypLE ExpressTM (Invitrogen, Waltham, MA, USA), resuspended in serum-free medium, and seeded into the upper chambers of the CIM-16 plates at a density of 3 × 10^4^ cells/well. After adding the cells, CIM-16 plates were incubated in the laminar flow hood for 30 min at room temperature allowing cells to settle before placing them in the RTCA DP instrument at 37 °C. Chemotactic migration values were obtained by subtracting background migration signals. At each condition measurements were performed in triplicate and analyzed for 12 h.

### 2.8. Proliferation Assay

Effects of modulation of *PLAU* expression on cells proliferation were evaluated by Trypan Blue Cell Analysis [48]. After 48 h of transient transfection, the MDA-MB-231 cells were harvested, counted, and re-plated in 1:4 ratio in a 24 well plate. Cell count determinations were repeated every 3–4 days until 144 h after transfection.

### 2.9. TCGA Analysis

The *PLAU* gene expression analysis was performed using R (version 4.2.1) and R studio (version 2022.07.2). RNA-Seq samples, from The Cancer Genome Atlas (TCGA), corresponding to TCGA-BRCA [49] dataset was obtained using R package TCGAbiolinks [50] (version 2.24.3). From the dataset, only samples that were categorized as “primary tumor” or “solid tissue normal” were used for analysis. In total, 1219 samples, comprising of 1106 “primary tumor” and 113 “solid tissue normal” samples were subjected to the analysis. The RNA-seq counts and the patient data were stored as DGEList() object of the edgeR package [51](version 3.38.4) to enable the use of different packages for the gene expression quantification. Subsequently, the RNA-seq counts were filtered and normalized using filterByExpr() and calcNormFactors() functions of the edgeR package (version 3.38.4). The filtered and normalized counts were further processed using voom() function of limma package [52] (version 3.52.4) to assess the expression of *PLAU* gene in primary tumor and solid tissue normal samples. The expression level of *PLAU* in the two sample categories were extracted as table, to perform statistical analysis and plot graphs using GraphPad prism (version 8.4.2). The higher expression of *PLAU* in primary tumor samples was statistically significant as determined by the Mann–Whitney test in GraphPad Prism (version 8.4.2).

### 2.10. Statistics

Statistical tests were performed using Graphpad Prism 7 software. Comparison between target conditions and controls were investigated with an unpaired two-tailed t-test. Differences were considered statistically significant if the *p*-value was <0.05. All data are presented as the mean ± S.D. of three independent, biological replicates, unless stated differently.

## 3. Results

### 3.1. Screening of sgRNAs for PLAU

The Cancer Genome Atlas (TCGA) data demonstrated a higher *PLAU* gene expression for 1095 breast cancer samples compared to 113 healthy breast tissue (Figure S1). To test the oncogenic activity of *PLAU* in breast cancer cells, twelve sgRNAs targeting the *PLAU* promoter were designed around the two alternative transcription start sites (TSSs) (see schematic representation in Figure 1), which are highly controlled by DNA methylation and histone methylation [28]. The sgOUTall (OUT1, 2, 3, 4) group is located outside of the CGI and upstream of the two alternative TSSs; the sgTSSall (TSS1, 2, 3, 4) group is located around TSS1; the sgINall (IN1, 2, 3, 4) group is located downstream of the TSSs and completely inside the CGI. Two additional mixtures were tested: sgMIX1 (TSS1, TSS3, IN2, IN4) and sgMIX2 (TSS2, TSS4, IN1, IN3) to more widely cover the promoter region.

**Figure 2:**
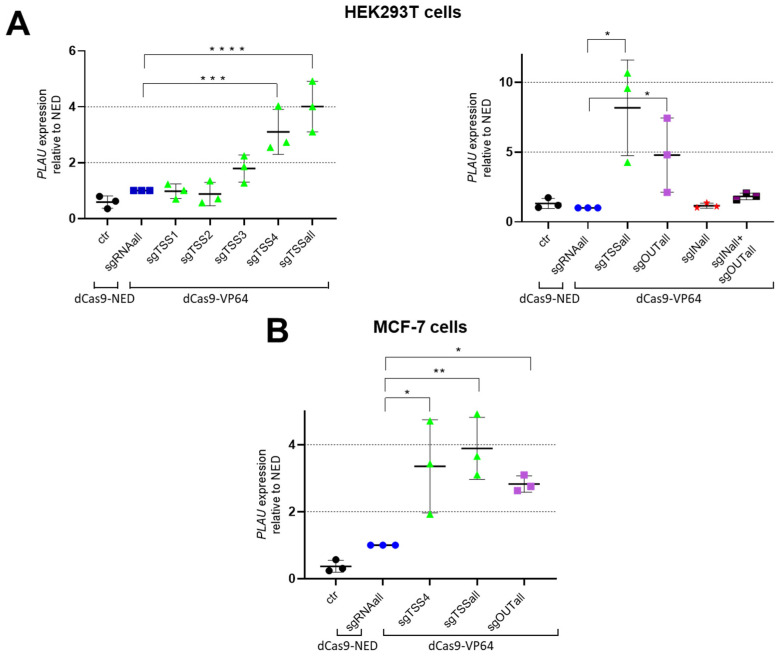
sgRNA-induced upregulation of *PLAU* expression in (**A**) HEK293T, and in (**B**) MCF-7 cells. (**A**) qRT-PCR results of HEK293T cells tested for VP64-induced upregulation using sgTSS4, individually and as mix, sgOUTall and/or sgINall; (**B**) qRT-PCR results of MCF-7 cells tested for VP64-induced upregulation using sgTSS4, sgTSSall and sgOUTall. Data are represented as means of 3 independent experiments, relative to *GAPDH* and normalised to transfected to cells with dCas9-NED. Significance is presented as compared to the dCas9-NED condition; * *p* < 0.05, ** *p* < 0.01, *** *p* < 0.005, **** *p* < 0.001.

These different sgRNAs were compared for their efficacy to modulate *PLAU* gene expression in transient transfection experiments in either MDA-MB-231 cells characterized by high *PLAU* levels, and in HEK293T and MCF-7 cell lines, which express *PLAU* at low levels (Figure 2). MDA-MB-231 cells were transfected with dCas9-SKD and CRISPRoff-v2.1 plasmids (Figure S2), whereas HEK293T and MCF-7 cells were transfected using dCas9-VP64 (Figure 2).

In MDA-MB-231 cells, sgTSS4, sgTSSall, sgMIX1 and sgMIX2 were tested two and five days after transfection. Only the sgTSSall group combined with dCas9-SKD or CRISPRoff-v2.1 resulted in reduced *PLAU* expression compared to dCas9-NED five days after transfection (Figure S2).

In HEK293T cells, in which the *PLAU* promoter is unmethylated, expression of dCas9-VP64 together with sgTSS4, or with the combination of four sgRNAs (sgTSSall) resulted in a 3.1-fold (*p* = 0.034) and 4.0-fold (*p* = 0.012) induction of the *PLAU* gene, respectively, relative to cells treated to express dCas9-NED (Figure 2A, left graph). A separate set of experiments confirmed the dCas9-VP64 induction with sgTSSall, leading to a 8.2-fold induction relative to dCas9-NED transfected cells (*p* = 0.021) (Figure 2A, right graph). In this set of experiments, the sgOUTall group resulted in a 4.8-fold induction of the *PLAU* gene relative to dCas9-NED (*p* = 0.022). No significant induction was obtained for the sgINall group or for a combination of sgINall and sgOUTall group.

In MCF-7 cells, where the *PLAU* promoter is hypermethylated (https://www.encodeproject.org/experiments/ENCSR000CPT/), sgTSS4 (the most effective guide from the sgTSSall group based on HEK293T results), sgTSSall, and sgOUTall, were tested for gene induction using dCas9-VP64 (Figure 2B). Both groups as well as the individual sgTSS4 resulted in a significant induction of the *PLAU* gene (3.9-fold for sgTSSall, 2.8-fold for sgOUTall and 3.4-fold for sgTSS4), relative to cells treated to express dCas9-NED. CRISPR-dCas9 off-targets effects were evaluated in MCF-7 upon transfections with plasmids transferring sgRNAs targeting a different gene (*KDM4A*) using dCas-VP64. No off-target regulation of *PLAU* was observed for dCas-VP64 in these cells (Figure S3).

### 3.2. Downregulation of the PLAU Gene

**Figure 3:**
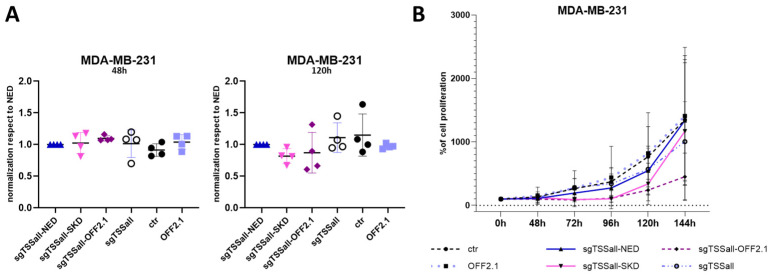
*PLAU* down-regulation and its functional effect in MDA-MB-231 cells. (**A**) qRT-PCR results of *PLAU* mRNA expression in MDA-MB-231 cells transfected to transiently express dCas9-SKD or CRISPRoff-v2.1 (OFF2.1) and sgTSSall at 2 (48 h, left panel) and 5 days (120 h) after transfection (right panel), compared to dCas9-NED. As negative controls, cells were transfected to express sgRNA only (sgTSSall) or CRISPRoff-v2.1 (OFF2.1) only. (**B**) Proliferation assay results of the cells transfected with plasmids shown in (**A**). Data are represented as means of 4 independent experiments.

Based on the obtained sgRNA screening results described above, we further investigated the functional effects of *PLAU* repression in MDA-MB-231. Co-transfecting sg*PLAU*tss1 with dCas9-SKD or with CRISPRoff-v2.1, did not result in significant difference after two days compared to the negative control (dCas9-NED) (Figure 3A left). Additionally, five days after transfection, sgTSSall-SKD and sgTSSall—OFF2.1 weakly reduced *PLAU* expression (means of 0.82 and 0.86-fold expression left, respectively, compared to cells expressing sgTSSall and dCas9-NED (Figure 3A, right panel)). Despite the variable, non-statistically significant reduction at mRNA level, we observed a clear decrease in cell proliferation for all CRISPRoff-v2.1 experiments at 144 h (Figure 3B). A complete block in cell growth was observed until 96 h for both sgTSSall-SKD and sgTSSall-OFFv2.1 expressing cells, but only CRISPRoff-v2.1, that contains both KRAB and DNMT3A/3L domains, was able to maintain reduced cell proliferation. These data indicate that mild *PLAU* downregulation can reduce the aggressiveness of the triple negative breast cancer cell.

In parallel, we adopted another approach to increase efficiency of *PLAU* gene repression. Stable MDA-MB-231 cells, engineered to express dCas9-NED, dCas9-G9A or its mutant, or -M.SssI-Q147L, a M.SssI variant with reduced DNA binding, or its catalytic inactive E186A mutant [53], were transduced a second time with lentiviral constructs containing either the four sgOUT(1–4), or a sgRNA without DNA recognizing insert (empty vector (EV)) as a control.

Even though a trend towards *PLAU* downregulation was achieved in MDA-MB-231 cells stably expressing -M.SssI-Q147L and G9A wild tipe and mutant for sgOUTall, no significant repression of *PLAU* could be observed (Figure S4).

### 3.3. Epigenetic Editing to Induce PLAU Upregulation

Next to growth inhibition upon repression, we set out to demonstrate the oncogenic activity of *PLAU* in cells in which *PLAU* is expressed at low levels by inducing *PLAU* expression using dCas9-VP64. Moreover, to investigate whether Epigenetic Editing could lead to long-term *PLAU* induction, the epigenetic enzymes PRDM9 (writing H3K4me3) and DOT1L (writing H3K79me), fused to CRISPR-dCas9, were tested using sgTSS4 and sgOUTall, in these cells (HEK293T and MCF-7 cells; Figure S5).

In all sets of experiments, *PLAU* induction using dCas9-VP64 was confirmed to be in the same range as shown in Figure 2 after 48 h (Figure S5A,B). Since VP64 is an artificial transcription factor without any enzymatic activity on its own, a transient effect was expected. Indeed, after 10 days of transfection the induction disappeared and *PLAU* expression became similar to control conditions (ctr and dCas9-NED).

Relative to cells treated to express dCas9-NED and the combination of guides, neither one of the epigenetic enzymes, nor a combination of both, was able to accomplish a significant induction of the *PLAU* gene, not in the short-term (48 h after transfection) nor in the long-term (10 days after transfection) experiments (Figure S5).

### 3.4. Functional Effects of PLAU Induction

**Figure 4:**
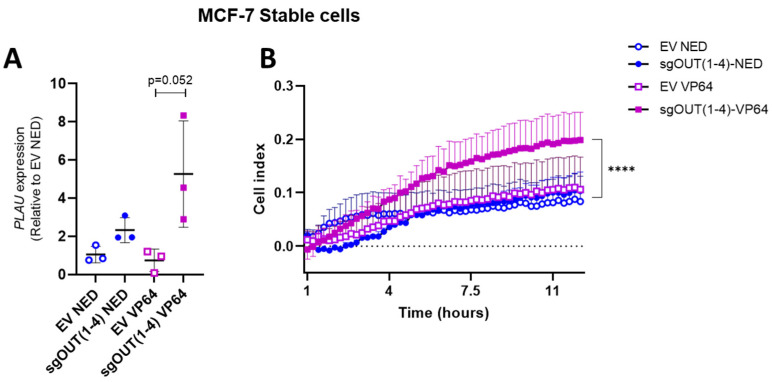
*PLAU* upregulation and its functional effect in stable MCF-7 cells. (**A**) qRT-PCR results of *PLAU* mRNA expression in stable MCF-7 cells using the sgOUT(1–4) or EV as negative control (**B**) Migration assay results of the cells transfected in (**A**). Data are represented as means of 3 independent experiments, relative to *GAPDH* and normalized to MCF-7-NED + EV; **** *p* < 0.001.

As *PLAU* is known to play an important role in breast cancer cell migration, it was investigated whether inducing *PLAU* expression alone was indeed sufficient to provoke functional changes in MCF-7 cells. To this end we analyzed the migratory capacities of the MCF-7 cells stably expressing dCas9-VP64 (MCF-7-VP64) after transient transfection with the sgOUT(1–4) plasmid, which expresses all four individual sgOUTall from a single plasmid. As negative controls, the MCF-7-NED stable cell line was transfected with the sgOUT(1–4) plasmid or empty guide vector (EV), which was also used to transfect the MCF-7-VP64 stable cells. After 48 h a 5.3-fold induction of *PLAU* expression was observed in MCF-7-VP64 cells transfected with sgOUT(1–4) compared to MCF-7-NED cells transfected with EV (*p* = 0.052) (Figure 4A). Twelve hours later, MCF-7-VP64 stable cells transfected with sg*PLAU* tandem displayed a 1.9-fold (*p* < 0.001) increase in migration compared to the MCF-7-NED stable cell line transfected with EV (Figure 4B) as measured by real-time analysis of transwell migration. These data indicate that our CRISPR-dCas9 system was able to influence the migratory capability of MCF-7 cells through upregulation of *PLAU* expression.

## 4. Discussion

Overexpression of *PLAU* has been identified as a promising therapeutic target for inhibition in (co)treatment of triple negative breast cancer in multiple studies [1,8,9,10], including ours [20,28]. Based on epigenetic studies, a role for DNA methylation has been suggested in inducing inhibition of *PLAU* expression [33,34,35]. Using CRISPR-dCas9 targeting tools, we aimed to reprogram *PLAU* gene expression in MDA-MB-231 and MCF-7 cells to validate its effects on the invasive proliferation and migratory properties. The versatility of the CRISPR-dCas9 platform to upregulate *PLAU* expression in cells expressing low levels, while repressing expression in *PLAU* overexpressing cells, strengthened our conclusion on the oncogenic function of *PLAU* and its involvement in breast tumor aggressiveness.

First, we showed that reduction in *PLAU* expression could be achieved in invasive hormone resistant MDA-MB-231 cells by CRISPRoff-v2.1, which decreased cancer cell proliferation. Conversely, an increase in *PLAU* expression was obtained in non-invasive, hormone-sensitive, ΕRα positive luminal A MCF-7 breast cancer cells by dCas9-VP64-sg TSSall leading to a higher migratory capacity of the cells.

Whilst WA treatment has been shown to provide promising therapeutic effects associated with a downregulation of *PLAU* [20], treatment with this phytopharmaceutical compound modulates the expression of multiple genes [24,27,28]. Gene targeted approaches such as CRISPR-dCas9-directed Epigenetic Editing thus assist in unravelling the contribution of individual WA-responsive genes in suppressing aggressive and invasive breast cancer phenotypes. For example, studies have shown effects of WA on FOXO3a and BIM mediated apoptosis, inhibition and induction of ROS [54,55,56,57]. An extensive microarray transcriptome profiling of MCF-7 and MDA-MB-231 cells identified multiple common, as well as cell line-specific target genes of WA, that were present in multiple pathways [20]. By targeting these genes, individually and in combination with CRISPR-dCas9-EDs, their specific involvement can be further investigated. This can ultimately assist in identification of potent therapeutic targets and contribute to more personalized treatment options. CRISPR-dCas9 targeting tools are well suited to target multiple genes at the same time, which is beneficial in conditions such as cancer where multiple genes are involved.

Although dCas9-VP64 is useful to up-regulate gene expression, it generally induces transient effects. Targeting of epigenetic writers or erasers to genes aims to achieve sustained gene expression effects. Moreover, such epigenetic editing tools can target multiple EDs to the same gene, which can result in additive, and potentially long-lasting effects. One example by Cano-Rodriguez et al. shows the additive effects of a combination of enzymes (PRDM9 (writing H3K4me3) and DOT1L (writing H3K79me)) in effectively re-expressing silenced genes. These effects were shown to be sustained when the promoter was hypomethylated [41]. Here, we used the same combination of enzymes to evaluate whether Epigenetic Editing could lead to long-term *PLAU* induction. However, no increase in *PLAU* expression was achieved when targeting PRDM9 or DOT1. The lack of gene induction observed when using sgTSS4 or sgOUTall in combination with PRDM9 and/or DOT1L as well as the limited repressive effects in MDA-MB-231 cells could be due to various factors, which become more pronounced for epigenetic effectors which likely have weaker transcriptional effects than the strong VP64 activator. Some of these factors are related to the intrinsic characteristics of the *PLAU* promoter (Figure 1). Data retrieved from Ensembl and USCS databases show that a CCCTC-binding factor (CTCF) binding site is present upstream of TSS1 which could affect binding of sgOUT1–4 from the sgOUTall group. These sites are well known transcription factor binding sites that can block the communication between enhancers and promoters [58], which could affect the efficiency of binding of sgRNAs. Alternatively, in Figure 1A phenotype-associated SNPs (Rs numbers) are depicted that are present in the *PLAU* promoter region. These are all regularly occurring SNPs, and it could be that specific SNPs in regions of sgRNA binding cause loss of binding affinity. Moreover, there are five possible splice-variants of *PLAU*, of which three are protein coding. The qRT-PCR primers were directed to the most validated and characterized isoform, which is also highly expressed in MDA-MB-231 cells. However, we did not investigate the impact of our epigenetic constructs on different transcript isoforms, which might be differentially affected, explaining the more pronounced functional effect in CRISPR-offv2.1 induced growth inhibition.

Interestingly, although targeting the recently described three-domain prolonged repressive dCas9 version (CRISPRoff-v2.1) did not improve the *PLAU* repression at the level of gene activity compared to targeting the KRAB domain alone on day 3, a functional improvement was obtained in cell growth inhibition measured after 6 days. In transiently transfected cells, *PLAU* expression reduction only occurred in the cells which express CRISPRoff-v2.1. It is conceivable that the failure of maintaining the reduced cell proliferation can be attributed to the cell fraction, in which the silencing of *PLAU* was not complete. Partial silencing is such that it does not induce cell death, but only a decrease in proliferation [59]. Another important factor that impacts the efficiency of Epigenetic Editing is delivery of large CRISPR-dCas9 constructs to the cells [60]. To efficiently deliver the CRISPR-dCas9 system to the cells, researchers make use of a variety of approaches. One option is to use lentiviruses, which integrate into the genome of the host cells, and in this way continuously express the desired constructs [61]. This approach can be useful to investigate mechanistic effects in vitro, however the phenomenon of integration, which can cause severe side effects such as cancer, limits its clinical use [62,63]. Another successful technique to deliver the Epigenetic Editing constructs into the cells are non-viral ligand-directed targeting approaches that target cancer cell specific surface receptors. These approaches, which might have therapeutic potential, make use of liposomes and other nanoparticles, that are more efficiently taken up by the cells, and are protected from degradation by the biological environment [62,64]. Further, as this method holds the possibility to selectively target disease tissues [65], it is more suitable to use in the clinic as side-effects caused by off-target effects are lowered and the uptake by the patient is improved compared to, e.g., viral-based methods [66]. However, a possible disadvantage is the shorter in vivo life-time some of these extracellular vesicles can have compared to adeno-associated virus (AAV) -mediated delivery [67,68]. This latter delivery method is the preferred option in clinical trials with gene editing therapies at the moment [60]. AAV is a non-pathogenic virus with a very mild immune response capable of delivering constructs in a preferred tissue, with a high efficiency and without integrating into the host genome [69,70]. Epigenetic Editing studies have successfully delivered CRISPR-dCas9-EDs in vivo using AAV delivery, although the limiting packaging capacity of these AAV vectors (~4.5 kb) and the large sizes of Epigenetic Editing constructs require optimization [71]. Extracellular vesicles (EVs) or ribonucleoprotein (RNP), based on the direct delivery of sgRNA and dCas protein, have been shown to be an interesting approach for therapeutic Epigenetic Editing [72].

Despite the many developments with regard to using CRISPR-dCas9 in gene expression modulation, the success of future clinical applications relies largely on safety and efficiency of delivery. The recent founding of various companies to translate Epigenetic Editing to the clinic will provide important insights on issues to be overcome to progress with Epigenetic Editing for cancer and beyond [72].

Indeed, our *PLAU*-modulation platform using dCas9-VP64 and CRISPRoff-v2.1 could be exploited further for use in a broad variety of pathological conditions that would benefit from *PLAU* modulation such as ischemic brain injury [73,74,75], lung fibrosis [76], male infertility [77], type 2 diabetes mellitus [78] and diabetic keratopathy [79], besides breast cancer [3,22]. The use of Epigenetic Editing tools, together with the optimization of in-patient delivery techniques, will open novel avenues to manipulate specific genes such as *PLAU* to treat aggressive breast cancer, and many other diseases.

## Data Availability

Not applicable.

## Supplementary Materials

The following supporting information can be downloaded at: www.mdpi.com/article/10.3390/biomedicines11010102/s1, Figure S1: Analysis of TCGA data assessing *PLAU* mRNA gene expression; Figure S2: Screening of sgRNAs to induce repression of *PLAU* expression in MDA-MB-231 cells; Figure S3: *PLAU* gene expression in MCF-7 cells transfected to express sgRNA targeting an irrelevant gene (*KDM4A*); Figure S4: Assessment of *PLAU* expression in MDA-MB-231 stable cell lines expressing repressing dCas9-EDs; Figure S5: Epigenetic Editing of *PLAU* in HEK293T cells and MCF-7 cells; Table S1: Primer sequences as used in the quantitative real-time PCR; Table S2: Plasmids used for transient and lentiviral delivery.

## Abbreviations

Bp	base pair
CGI	CpG island
CRISPR	clustered regularly interspaced short palindromic repeats
DMEM	Dulbecco’s modified Eagle medium
DNA	deoxyribonucleic acid
ECM	extracellular matrix
ED	effector domain
FBS	fetal bovine serum
HEK293T	human embryonic kidney cells
MCS	multicloning site
NED	no effector domain
PEI	polyethylenimine
*PLAU*	plasminogen activator, urokinase
sgRNA	single guide ribonucleic acid
SKD	super KRAB domain
u-PA	plasminogen activator
WA	withaferin A
