# Supplementary material for: Functional Validation of the Putative Oncogenic Activity of PLAU
Source: Biomedicines. 2022 Dec 30;11(1):102. doi: 10.3390/biomedicines11010102 (PMC9856075; doi:10.3390/biomedicines11010102)
Supplement: Supplementary file 1 [file biomedicines-11-00102-s001.zip › biomedicines-2087153-supplementary.pdf]

Supplementary Materials

Functional Validation of the Putative Oncogenic Activity of *PLAU*

Federica Sarno [1], Désirée Goubert [1], Emilie Logie [2], Martijn G. S. Rutten [1,3], Mihaly Koncz [4,5,6], Christophe Deben [7], Anita E. Niemarkt [1], Lucia Altucci [8,9,10], Pernette J. Verschure [4], Antal Kiss [5], Wim Vanden Berghe [2] and Marianne G. Rots [1,*]

[1] Epigenetic Editing, Department of Pathology and Medical Biology, University Medical Centre Groningen, University of Groningen, Hanzeplein 1, 9713 GZ Groningen, The Netherlands
[2] Center for Oncological Research (CORE), Integrated Personalized & Precision Oncology Network (IPPON), Laboratory of Protein Chemistry, Proteomics and Epigenetic Signalling (PPES), Department of Biomedical Sciences, University of Antwerp, 2610 Wilrijk, Belgium
[3] Department of Paediatrics, University Medical Centre Groningen, University of Groningen, Hanzeplein 1, 9713 GZ Groningen, The Netherlands
[4] Swammerdam Institute for Life Sciences, University of Amsterdam, Science Park 904, 1098 XH Amsterdam, The Netherlands
[5] Institute of Biochemistry, Biological Research Centre, H-6726 Szeged, Hungary
[6] Doctoral School of Biology, Faculty of Science and Informatics, University of Szeged, H-6726 Szeged, Hungary
[7] Center for Oncological Research (CORE), Integrated Personalized & Precision Oncology Network (IPPON), University of Antwerp, 2610 Wilrijk, Belgium
[8] Department of Precision Medicine, Universita' degli Studi della Campania "Luigi Vanvitelli", 80138 Naples, Italy
[9] Biogem Institute of Molecular and Genetic Biology, 83031 Ariano Irpino, Italy
[10] IEOS Istituto per l'Endocrinologia e l'Oncologia "Gaetano Salvatore", Via Pansini, 80131 Napoli, Italy
* Correspondence: m.g.rots@umcg.nl

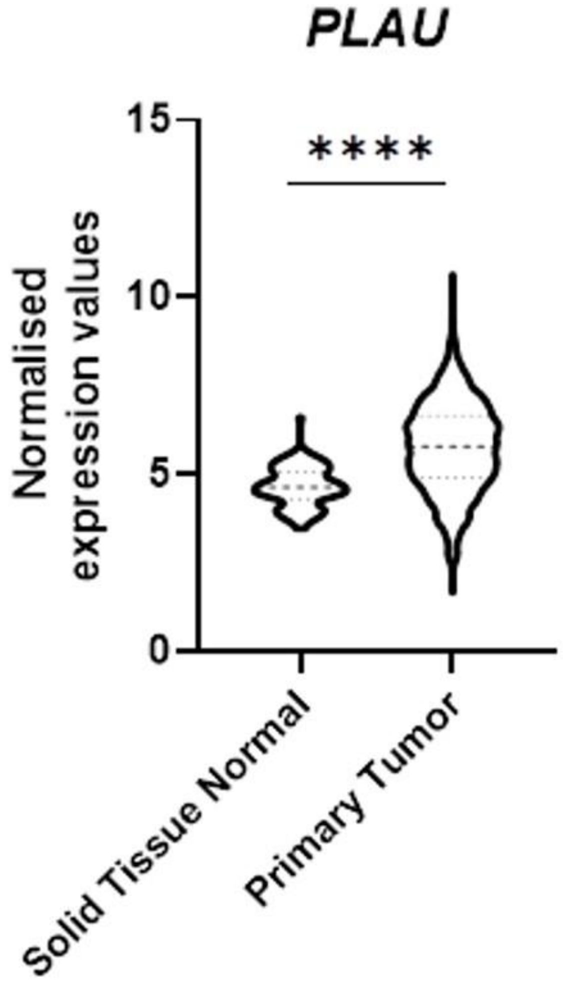

Figure S1. Analysis of TCGA data assessing *PLAU* mRNA gene expression in 113 healthy breast tissues compared to 1095 breast cancer samples. Lower expression of *PLAU* was observed in healthy tissues compared to breast cancer samples. Statistical significance was evaluated by Mann-Whitney test (* * * * $p < 0.0001$).

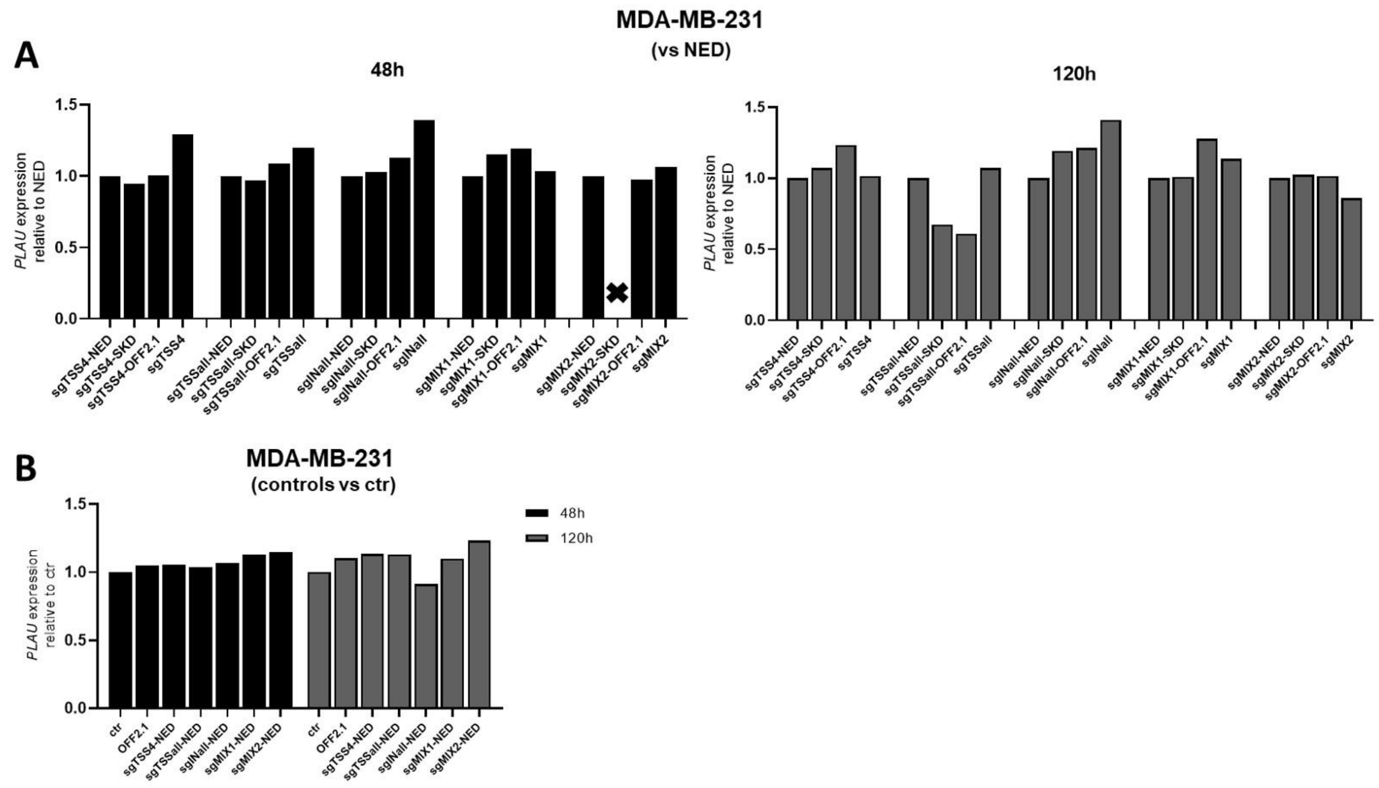

Figure S2. Screening of sgRNAs to induce repression of *PLAU* expression in MDA-MB-231 cells. (A) qRT-PCR of *PLAU* mRNA expression in transiently transfected MDA-MB-231 cells with plasmids expressing for sgTSS4, sgTSSall, sgINall, sgMIX1 or sgMIX2 together with dCas9-SKD or CRISPRoff-v2.1. Cells were harvested 2 days (48h, left) and 5 days (120h, right) after transfection. Results are represented as *PLAU* expression fold change with respect to cells transfected with the dCas9-NED (n=1). (B) Control qRT-PCR experiments of *PLAU* mRNA expression in transient transfected MDA-MB-231 cells expressing sgTSS4, sgTSSall, sgINall, sgMIX1 or sgMIX2 together with dCas9-NED compared to control cells (transfected with an "empty "guide RNA plasmid (ctr)) and CRISPRoff-v2.1 transfected without sgRNA compared to ctr (n=1).

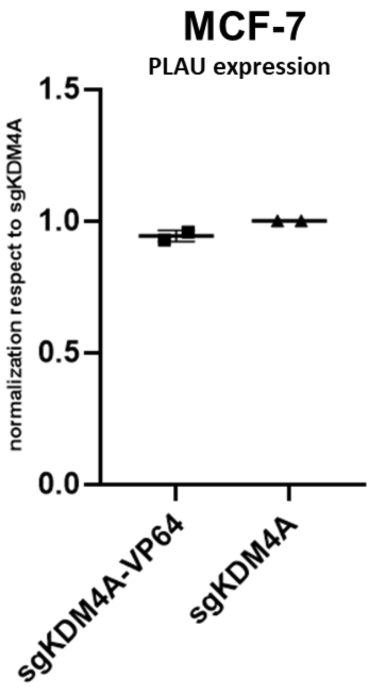

Figure S3. *PLAU* gene expression in MCF-7 cells transfected to express sgRNA targeting an irrelevant gene (*KDM4A*). qRT-PCR results of *PLAU* mRNA expression in MCF-7 cells co-transfected to transiently express dCas9-VP64 with sgRNA targeting *KDM4A* (sgKDM4A1-6) compared with cells expressing sgKDM4A1-6-only (48h after transfection).

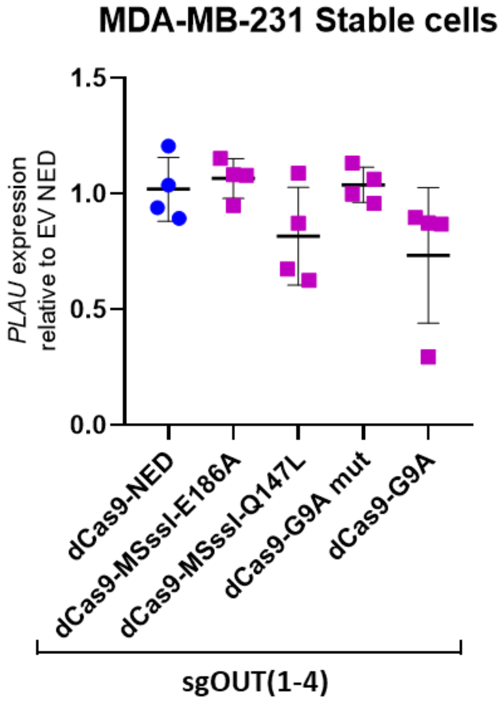

Figure S4. Assessment of *PLAU* expression in MDA-MB-231 stable cell lines expressing repressing dCas9-EDs. Results obtained by qRT-PCR. Cells were first transduced with lentiviral dCas9-EDs and selected with puromycin, after which the stable cells were transduced a second times with lentiviral sgOUT(1-4) or EV control. Data are represented as means of 4 independent experiments, relative to GAPDH and normalised to MDA-MB-231-NED + EV.

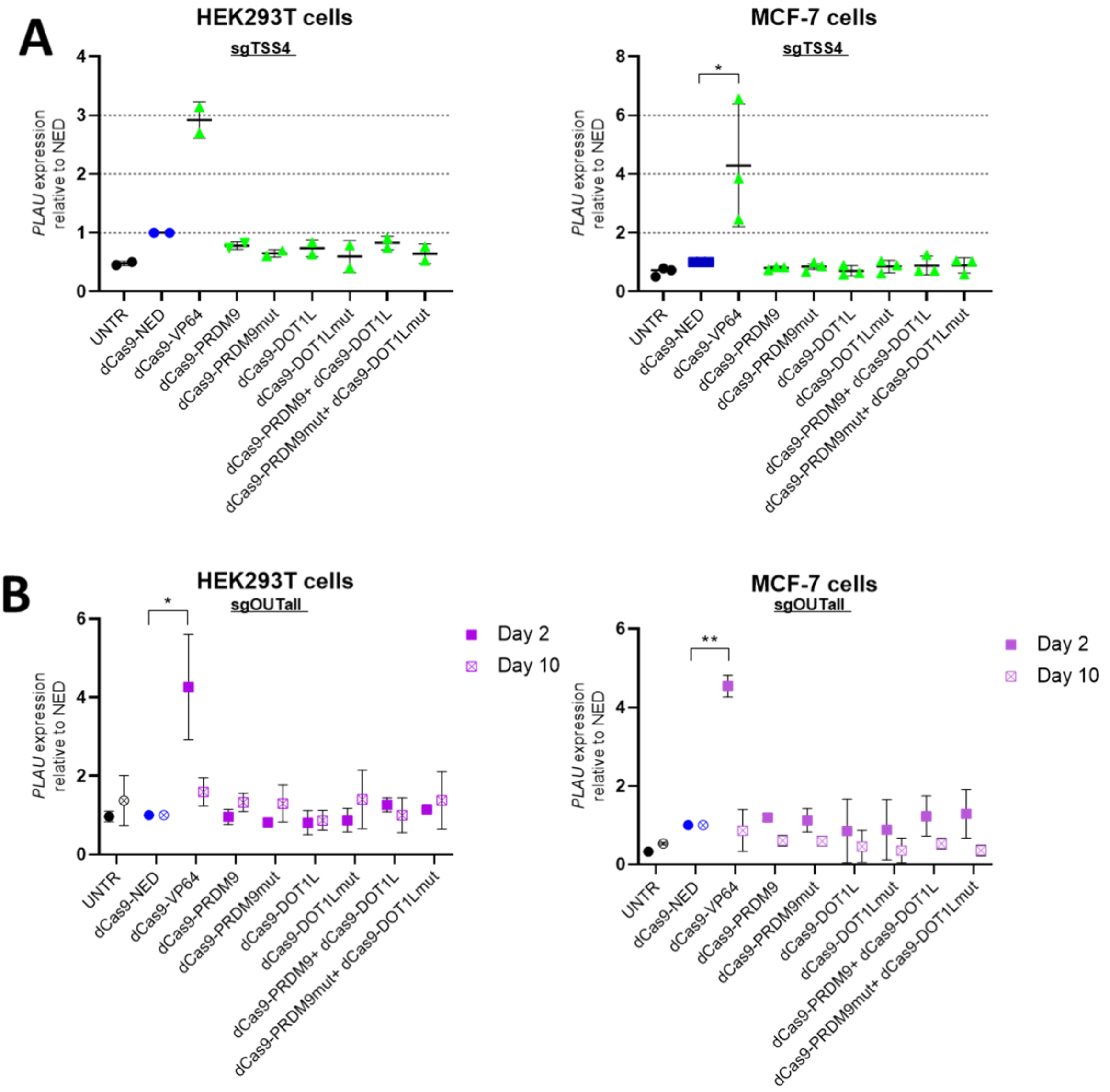

Figure S5. Epigenetic Editing of *PLAU* in HEK293T cells and MCF-7 cells. (A) qRT-PCR of *PLAU* mRNA expression in HEK293T and MCF-7 cells tested with sgTSS4 and epigenetic enzymes PRDM9 and DOTL1L, dCas9-VP64 was used as positive control (B) qRT-PCR of *PLAU* mRNA expression in HEK293T and MCF-7 cells tested for sgOUTall with epigenetic enzymes PRDM9 and DOTL1L. Cells were harvested 2 days and 10 days after transfection, dCas9-VP64 was used as positive control. Data are represented as means of 2 (HEK293T in A) or 3 independent experiments (MCF-7 in A and both cell lines in B), normalised to *GAPDH* and *PLAU* fold change relative to cells treated dCas9-NED; * $p < 0.05$ ** $p < 0.01$. With sgTSS4 an induction of 3.0-fold compared to dCas9-NED was obtained in HEK239T cells, and a 4.3-fold induction compared to dCas9-NED was achieved in MCF-7 cells ($p = 0.048$ compared to dCas9-NED) (Figure S5 A). With sgOUTall a 4.3-fold induction was seen in HEK239T ($p = 0.033$ compared to dCas9-NED) cells and 4.6-fold compared to dCas9-NED in MCF-7 cells ($p = 0.002$ compared to dCas9-NED) (Figure S5 B). In MCF-7 cells, all Epigenetic Editing constructs as well as their mutants together with sgOUTall showed a slight induction when measured after 48 hours compared to cells treated with transfection reagent only (UNTR). This might be explained by dCas9 binding (e.g. steric hindrance at this location) and was indeed also observed for cells expressing dCas9-NED in Figure S5.

Table S1. Primer sequences as used in the quantitative real-time PCR.

Primer Sequences	
**PLAU**	Fw: GACTCCAAAGGCAGCAATGAA<br>Rv: GTGCTGCCCTCCGAATTTCT
**GAPDH**	Fw: CCACATCGCTCAGACACCAT<br>Rv: GCGCCCAATACGACCAAAT

Table S2. Plasmids used for transient and lentiviral delivery.

**PLASMIDS**
MLM3705 (Addgene #47754)
dCas9-VP64
dCas9-NED
dCas9-PRDM9 and dCas9-PRDM9mut (mutant form)
dCas9-DOT1L and dCas9-DOT1Lmut (mutant form)
CRISPRoff-v2.1 (Addgene #167981)
MLM3636 (Addgene #43860)
sgOUT1
sgOUT2
sgOUT3
sgOUT4
sgTSS1
sgTSS2
sgTSS3
sgTSS4
sgIN1
sgIN2
sgIN3
sgIN4
sgKDM4A1
sgKDM4A2
sgKDM4A3
sgKDM4A4
sgKDM4A5
sgKDM4A6
pHAGE EF1α dCas9-VP64 (Addgene plasmid #50918)
pCMVΔR8.91
pCMV-VSV-G (#8454, Addgene)
pMLM2.0 (from sgRNA(MS2)_zeo backbone; Addgene#61427)
sgOUT(1-4) (sgOUT1+ sgOUT2+ sgOUT3+ sgOUT4)
